# Thermodynamic insight into the growth of nanoscale inclusion of Al-deoxidation in Fe–O–Al melt

**DOI:** 10.1038/s41598-020-73317-4

**Published:** 2020-10-09

**Authors:** Hong Lei, Yuanyou Xiao, Guocheng Wang, Hongwei Zhang, Wei Jin, Lifeng Zhang

**Affiliations:** 1 Key Laboratory of Electromagnetic Processing of Materials, Ministry of Education, Northeastern University, Shenyang, 110819 Liaoning Province P. R. China; 2 Key Laboratory of Chemical Metallurgy Engineering Liaoning Province, University of Science and Technology Liaonin, Anshan, 114051 Liaoning P. R. China; 3 Key Laboratory of Synthetic and Biological Colloids, Ministry of Education, School of Chemical and Material Engineering, Jiangnan University, Wuxi, 214122 Jiangsu Province P. R. China; 4 College of Materials Science and Engineering, Yanshan University, Qinhuangdao, 066004 Hebei P. R. China

**Keywords:** Chemistry, Materials science

## Abstract

Products of Al-deoxidation reaction in iron melt are the most common inclusions and play an important effect on steel performance. Understanding the thermodynamics on nano-alumina (or nano-hercynite) is very critical to explore the relationship between Al-deoxidation reaction and products growth in iron melt. In present study, a thermodynamic modeling of nano-alumina inclusions in Fe–O–Al melt has been developed. The thermodynamic results show that the Gibbs free energy changes for the formation of nano-Al_2_O_3_ and nano-FeAl_2_O_4_ decrease with the increasing size and increase with the increasing temperature. The Gibbs free energy changes for transformation of nano-Al_2_O_3_ into bulk-Al_2_O_3_ increase with the increasing size and temperature. The thermodynamic curve of nano-alumina (or nano-hercynite) and the equilibrium curve of bulk-alumina (or bulk-hercynite) obtained in this work are agree with the published experimental data of Al-deoxidation equilibria in liquid iron. In addition, the thermodynamic coexisting points about Al_2_O_3_ and FeAl_2_O_4_ in liquid iron are in a straight line and coincide with the various previous data. It suggested that these scattered experimental data maybe in the different thermodynamic state of Al-deoxidized liquid iron and the reaction products for most of the previous Al-deoxidation experiments are nano-alumina (or nano-hercynite).

## Introduction

Alumina inclusions are harmful to the quality of the steel production, because of their high melting point and high hardness. In order to reduce and eliminate the harm of alumina inclusions, the steelmakers try their best to remove all inclusions, or transform the solid alumina to liquid (or partially liquid) calcium aluminates by Ca treatment^1,2^. On the other hand, they want to make full use of some special inclusions to improve the steel performance^3–6^. For example, the fine inclusions can be utilized as nucleation sites and make positive contribution to the nucleation of acicular ferrite^3–6^. From this point of view, refining of alumina inclusions can be one of the important way to enhance the quality of steel products. To refine the size of alumina inclusions, it is necessary to study the formation process of alumina and the thermodynamics on nanoscale inclusion during Al-deoxidation in molten steel.

In order to investigate the evolution process of alumina inclusion in liquid iron, several researchers^7–9^ carried out their experiments of Al-deoxidation in iron melt by ultra-rapid cooling method. Zhao et al.^7^ carried out the Al-deoxidation experiments in Fe–O melt by ultra-rapid cooling methods and found the shapes of alumina inclusions are small and irregular in various experimental samples. They reported that the equivalent radius of alumina inclusions are between 15 and 150 nm^7^. Wasai et al.^8^ also found a series of small alumina from nanoscale to microscale in Al-deoxidation experiments by this methods. It can be suggested that nano-alumina is the intermediate product of the crystallization of bulk alumina inclusions during Al-deoxidation process. Thus, the thermodynamics of nano-alumina in liquid iron is the basic to investigate the relationship of the size of alumina inclusions and Al-deoxidation reaction. Thermodynamic for Al-deoxidation in liquid iron have been extensively studied since the middle of twentieth century^10–30^. However, most of the researchers are focused on the thermodynamic equilibrium between the bulk-alumina and iron melt, while less known about the thermodynamic properties of nano-alumina in liquid iron. Many previous work proved that the thermodynamic properties of the nano-alumina are different from that of the bulk-alumina, and the thermodynamic difference among the nanoscale inclusions is more obvious with the decreasing size of inclusions^31–38^. It was reported that the interfacial free energy between nano-alumina and liquid iron are decreased with the decreasing of the size of alumina, and the Gibbs free energy change of Al-deoxidation reaction has very close relation with the size change of alumina inclusion^31^. Wang et al.^32–34^ also reported that the thermodynamics of Al-deoxidation reaction in liquid iron is dependent on the size of alumina inclusions. Therefore, a thermodynamic modeling about nano-alumina in liquid iron is necessary.

In this paper, the thermodynamic modeling for Al-deoxidation reaction between nano-alumina inclusions and liquid iron has been developed. Base on the density functional theory (DFT) calculations, the thermodynamic curves and coexisting points corresponding to various size alumina and hercynite inclusions in liquid iron have been obtained. The effect of products size on Al-deoxidation equilibrium thermodynamics have been discussed.

## Theoretical calculation

### Thermodynamic modeling of nano-Al_2_O_3_ in Fe–O–Al melt

**Figure 1:**
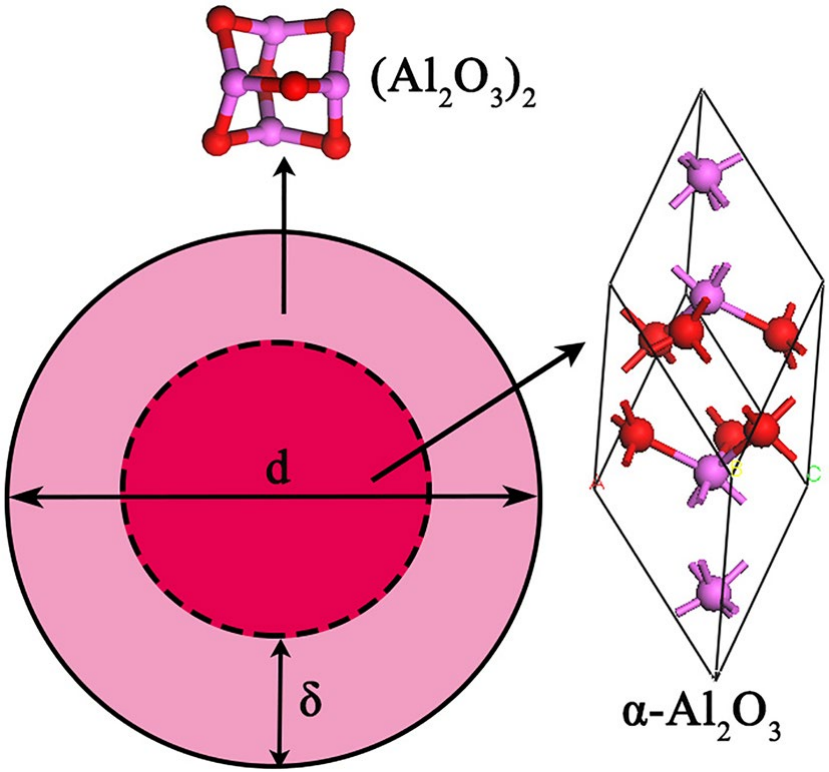
Structure of nano-Al_2_O_3_.

The nano-Al_2_O_3_ consists of two parts^38,39^, an internal part that the atoms located in the lattice of Al_2_O_3_ crystal and an external part that the atoms situated in the surface layer of Al_2_O_3_ particle. Thus, the calculations of thermodynamic properties of nano-Al_2_O_3_ should be dealt with separately. The total Gibbs free energy of nano-Al_2_O_3_ *G* can obtained as^38,39^

1 $$ {\text{G}} = (1{\text{ } - \text{ x}}_{s} ){\text{G}}_{i} + {\text{x}}_{s} {\text{G}}_{s} $$

where *G*_s_ and *G*_i_ are the Gibbs free energy of the external part and internal part of nano-Al_2_O_3_, *x*_s_ is the atomic fractions in the surface of nano-Al_2_O_3_. As shown in Fig. 1, the nano-Al_2_O_3_ described as a sphere particle with diameter *d*, contains a shell of *δ* thickness and a core with diameter (*d* − 2*δ*). The atomic fractions *x*_s_ in the surface of nano-Al_2_O_3_ can be obtained as

2 $$ {\text{x}}_{s} = \frac{{{\text{N}}_{s} }}{{{\text{N}}_{s} + {\text{N}}_{i} }} = \frac{{{\text{V}}_{s} {\uprho }_{s} }}{{{\text{V}}_{i} {\uprho }_{i} + {\text{V}}_{s} {\uprho }_{s} }} = \frac{{1 - \left( {1 - \frac{{2{\updelta }}}{{\text{d}}}} \right)^{3} }}{{1 + \left( {\frac{{{\uprho }_{i} }}{{{\uprho }_{s} }} - 1} \right)\left( {1 - \frac{{2{\updelta }}}{{\text{d}}}} \right)^{3} }} $$

where the subscripts *i* and *s* stand for the internal part or the surface part of nano-Al_2_O_3_, *N* is the atom numbers, *V* is the volumes, *ρ* is the atomic densities.

**Figure 2:**
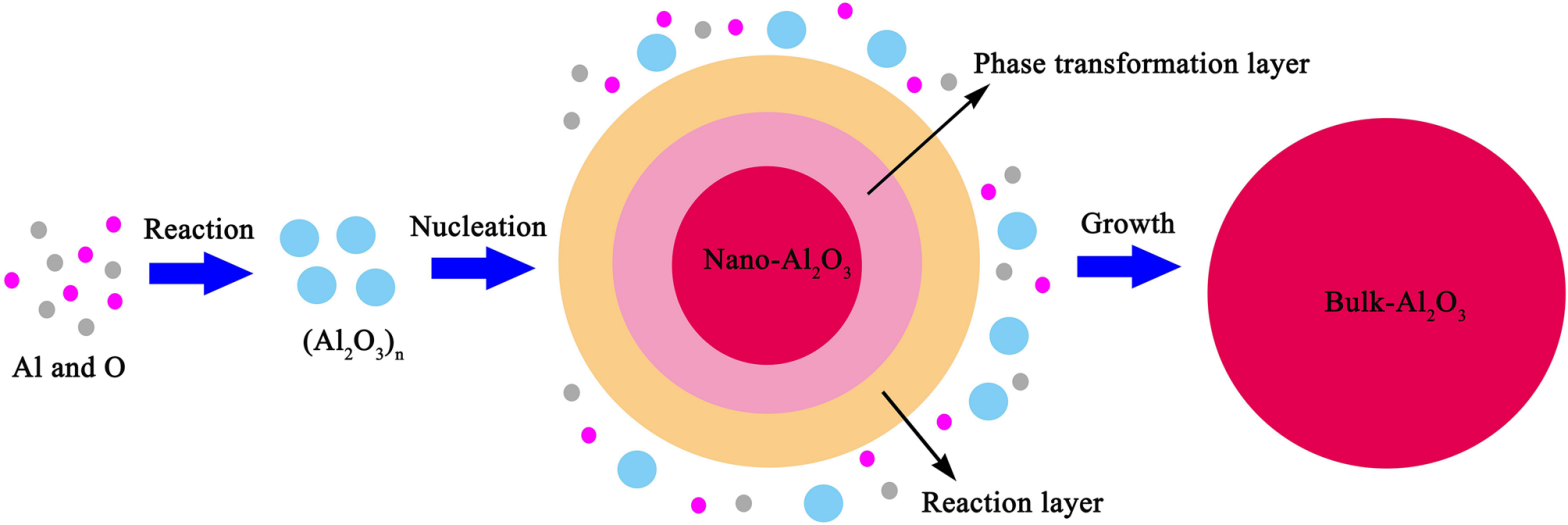
Schematic of the nucleation and growth of Al_2_O_3_ inclusion.

Some experiments reported that the atomic density of nano-particle surface is lower than that of the perfect crystal by 10–30%^39,40^. Thus, the value of *ρ*_i_/*ρ*_s_ was taken as 1.2 in the present calculation. It was reported that the nanocrystalline surface is short-range order structure and there exist a liquid-like structure layer on the surface of nano-particle^41–43^. As can be seen from Fig. 2, nano-Al_2_O_3_ is the intermediate of Al_2_O_3_ product particle growth in the Al-deoxidation reaction, and its surface is formed by the aggregation and phase transformation of (Al_2_O_3_)_n_ clusters. Therefore, it is logical to conclude that the surface structure of the nano-Al_2_O_3_ is a short-range order structure and is similar to the structure of (Al_2_O_3_)_n_ clusters. Furthermore, some studies reported that the surface of nano-particle usually contains two or three atom layers^5,44^. In this work, for simplicity, the (Al_2_O_3_)_2_ cluster, which contains three-atom thick, was used to describe the surface structure of nano-Al_2_O_3_, and the α-Al_2_O_3_ crystal was used to describe the internal part structure of nano-Al_2_O_3_ as show in Fig. 1. Thus, the Gibbs free energy of the internal part of nano-Al_2_O_3_ equal to the Gibbs free energy of α-Al_2_O_3_ crystal, and the Gibbs free energy of the external part of nano-Al_2_O_3_ equal to the Gibbs free energy of (1/2) (Al_2_O_3_)_2_. Moreover, the length of Al–O bond for alumina cluster is about 0.17 nm^34^. In other words, the thickness of three atom layers is about 0.5 nm. In this work, the thickness of the nano-Al_2_O_3_ surface *δ* was taken as 0.5 nm in calculation. The atomic fraction of the shell components are shown in Table 1.

**Table 1:** Atomic fractions of the surface components.

*d* (nm)	2	3	4	5	6	8
*x*_s_ (%)	85.37	66.43	53.31	44.27	37.76	29.11
*d* (nm)	10	20	40	60	100	200
*x*_s_ (%)	23.65	12.17	6.17	4.13	2.49	1.25

### Calculation methods

The Gibbs free energy of Al_2_O_3_ clusters and α-Al_2_O_3_ crystal were calculated by DFT method. During the calculations, the generalized-gradient approximation of Perdew-Burke-Ernzerhof (PBE) was applied as the exchange–correlation potential function^46^. The initial structures of (Al_2_O_3_)_2_ cluster and α-Al_2_O_3_ crystal were selected from the previous work^33^. The Gibbs free energy of (Al_2_O_3_)_2_ cluster and α-Al_2_O_3_ crystal were calculated by the equations^33–37^

3 $$ G = E + H - TS $$

where *E* is the total energy of alumina cluster or crystal at 0 K; *H* and *S* are the enthalpy and entropy of alumina cluster or crystal, respectively. The *H* and *S* were obtained by the analysis of atomic harmonic vibrational frequency of alumina cluster or crystal, which are the functions of temperature *T*. The calculation details of the relationship among the atomic harmonic vibrational frequency, the thermodynamic properties and temperature can be found in our previous study^33–37^.

### Gibbs free energy changes for the formation of nano-Al_2_O_3_ in liquid iron

The reaction equation of Al-deoxidation in Fe–O–Al melt can be written as:

4 $$ {2}\left[ {{\text{Al}}} \right] \, + { 3}\left[ {\text{O}} \right] \, = {\text{ Al}}_{{2}} {\text{O}}_{{3}} \left( {{\text{bulk}}} \right) $$

Nano-alumina is the intermediate product of the formation of bulk alumina inclusion and can be formed at first during Al-deoxidation process. The formation of nano-Al_2_O_3_ in liquid iron can be described as

5 $$ {2}\left[ {{\text{Al}}} \right] \, + { 3}\left[ {\text{O}} \right] \, = {\text{ nano } - \text{ Al}}_{{2}} {\text{O}}_{{3}}. $$

Then, nano-Al_2_O_3_ continue to grow up into stable bulk α-Al_2_O_3_ inclusions, and expressed as

6 $$ {\text{nano } - \text{ Al}}_{{2}} {\text{O}}_{{3}} \to {\text{ Al}}_{{2}} {\text{O}}_{{3}} \left( {{\text{bulk}}} \right). $$

The Gibbs free energy change of the formation of nano-Al_2_O_3_ as Eq. (5) is $\Delta {\text{G}}_{F}^{\theta }$, and the Gibbs free energy change for the transformation of nano-Al_2_O_3_ into bulk-Al_2_O_3_ as Eq. (6) is $\Delta {\text{G}}_{T}^{\theta }$. Based on Eqs. (4)–(6), $\Delta {\text{G}}_{F}^{\theta }$ and $\Delta {\text{G}}_{T}^{\theta }$ for the formation and transformation of different size nano-Al_2_O_3_ in iron melt can be calculated as

7 $$ \Delta {\text{G}}_{F}^{\theta } = \Delta {\text{G}}_{{Al_{2} O_{3} (bulk)}}^{\theta } { - }\Delta {\text{G}}_{T}^{\theta } $$

8 $$ \Delta {\text{G}}_{T}^{\theta } = {\text{G}}_{{Al_{2} O_{3} (bulk)}} {\text{ } - \text{ G}}_{{nano{ - }Al_{2} O_{3} }} $$

where $\Delta {\text{G}}_{{Al_{2} O_{3} (bulk)}}^{\theta }$ is Gibbs free energy change of the formation of α-Al_2_O_3_(bulk) as Eq. (4), ${\text{G}}_{{Al_{2} O_{3} (bulk)}}$ is Gibbs free energy of α-Al_2_O_3_ (bulk), ${\text{G}}_{{nano{ - }Al_{2} O_{3} }}$ is Gibbs free energy of nano-Al_2_O_3_. The value of $\Delta {\text{G}}_{{Al_{2} O_{3} (bulk)}}^{\theta }$ recommended by JPSP^10^ was used in the calculations as: $\Delta {\text{G}}_{{Al_{2} O_{3} (s)}}^{\theta }$ = − 1,225,000 + 393.8* T* J/mol.

**Figure 3:**
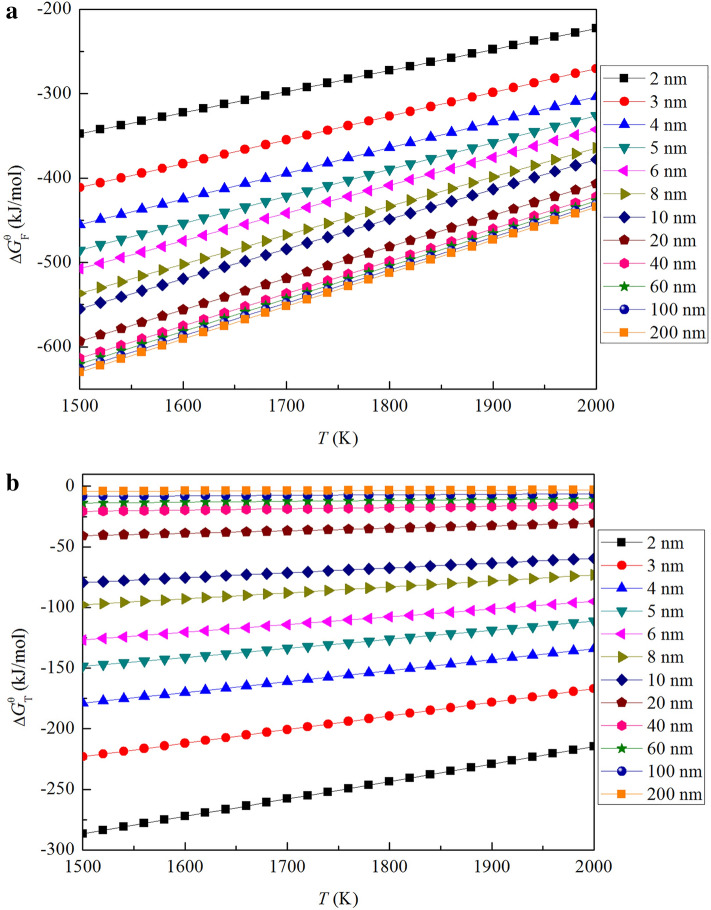
Gibbs free energy changes for the formation and transformation of nano-Al_2_O_3_ in liquid iron at 1873 K. (**a**) $\Delta {\text{G}}_{F}^{\theta }$, (**b**) $\Delta {\text{G}}_{T}^{\theta }$.

Figure 3 show the value of $\Delta {\text{G}}_{F}^{\theta }$ and $\Delta {\text{G}}_{T}^{\theta }$ (from 1500 to 2000 K). It can be seen from Fig. 3, $\Delta {\text{G}}_{F}^{\theta }$ decreases with the increasing size of nano-Al_2_O_3_, while $\Delta {\text{G}}_{T}^{\theta }$ increases with the increasing size of nano-Al_2_O_3_. It implies that the thermodynamic driving energy for the formation of nano-Al_2_O_3_ increases gradually with the increasing of alumina size, while the thermodynamic driving energy for transformation of nano-Al_2_O_3_ into bulk-Al_2_O_3_ decreases gradually with the increasing of alumina size. On the other hand, the value of $\Delta {\text{G}}_{F}^{\theta }$ and $\Delta {\text{G}}_{T}^{\theta }$ increase with the increasing temperature. This result indicates that both the formation of nano-Al_2_O_3_ and the transformation of nano-Al_2_O_3_ into bulk-Al_2_O_3_ at the low temperature is more easier than that at the high temperature.

### Gibbs free energy changes for the formation of nano-FeAl_2_O_4_ in liquid iron

Hercynite (FeAl_2_O_4_) is one of the product of Al-deoxidation reaction in liquid iron when [Al] at low content and [O] at high content. The equilibrium between FeAl_2_O_4_ and Fe–O–Al melt can be expressed as

9 $$ {\text{Fe }}\left( {\text{l}} \right) \, + { 4}\left[ {\text{O}} \right] \, + { 2}\left[ {{\text{Al}}} \right] \, = {\text{ FeAl}}_{{2}} {\text{O}}_{{4}} \left( {\text{s}} \right). $$

The activity of Fe(l) and FeAl_2_O_4_(s) is unity, and the activity of oxygen and aluminum is approximately its weight percent concentration because of [% Al] < 0.1 and [% O] < 0.1 in the liquid iron. Thus, the equilibrium constant ${\text{K}}_{{FeAl_{2} O_{4} }}$ for Eq. (10) can be expressed as follows

10 $$ \ln {\text{K}}_{{FeAl_{2} O_{4} }} = - \frac{{\Delta {\text{G}}_{{FeAl_{2} O_{4} }}^{\theta } }}{{R{\text{T}}}} = - 4\ln \alpha_{[O]} - 2\ln \alpha_{[Al]} = - 4\ln [\% O] - 2\ln [\% Al] $$

where α_i_ are the activity of element Al and O in the liquid iron, $\Delta {\text{G}}_{{FeAl_{2} O_{4} }}^{\theta }$ is Gibbs free energy changes for the formation of FeAl_2_O_4_ in liquid iron and can be calculate by Eqs. (4) and (11)

11 $$ {\text{Fe}}\left( {\text{l}} \right) \, + \, \left[ {\text{O}} \right] \, + {\text{ Al}}_{{2}} {\text{O}}_{{3}} \left( {\text{s}} \right) \, = {\text{ FeAl}}_{{2}} {\text{O}}_{{4}} ,\,\log {\text{K}} = - 2.84 + 7640/T^{11}. $$

Nano-FeAl_2_O_4_ is the intermediate product of the formation of bulk FeAl_2_O_4_ inclusion during Al-deoxidation process, and the formation of nano-FeAl_2_O_4_ can be described as

12 $$ {\text{Fe }}\left( {\text{l}} \right) \, + { 4}\left[ {\text{O}} \right] \, + { 2}\left[ {{\text{Al}}} \right] \, = {\text{ nano } - \text{ FeAl}}_{{2}} {\text{O}}_{{4}}. $$

**Figure 4:**
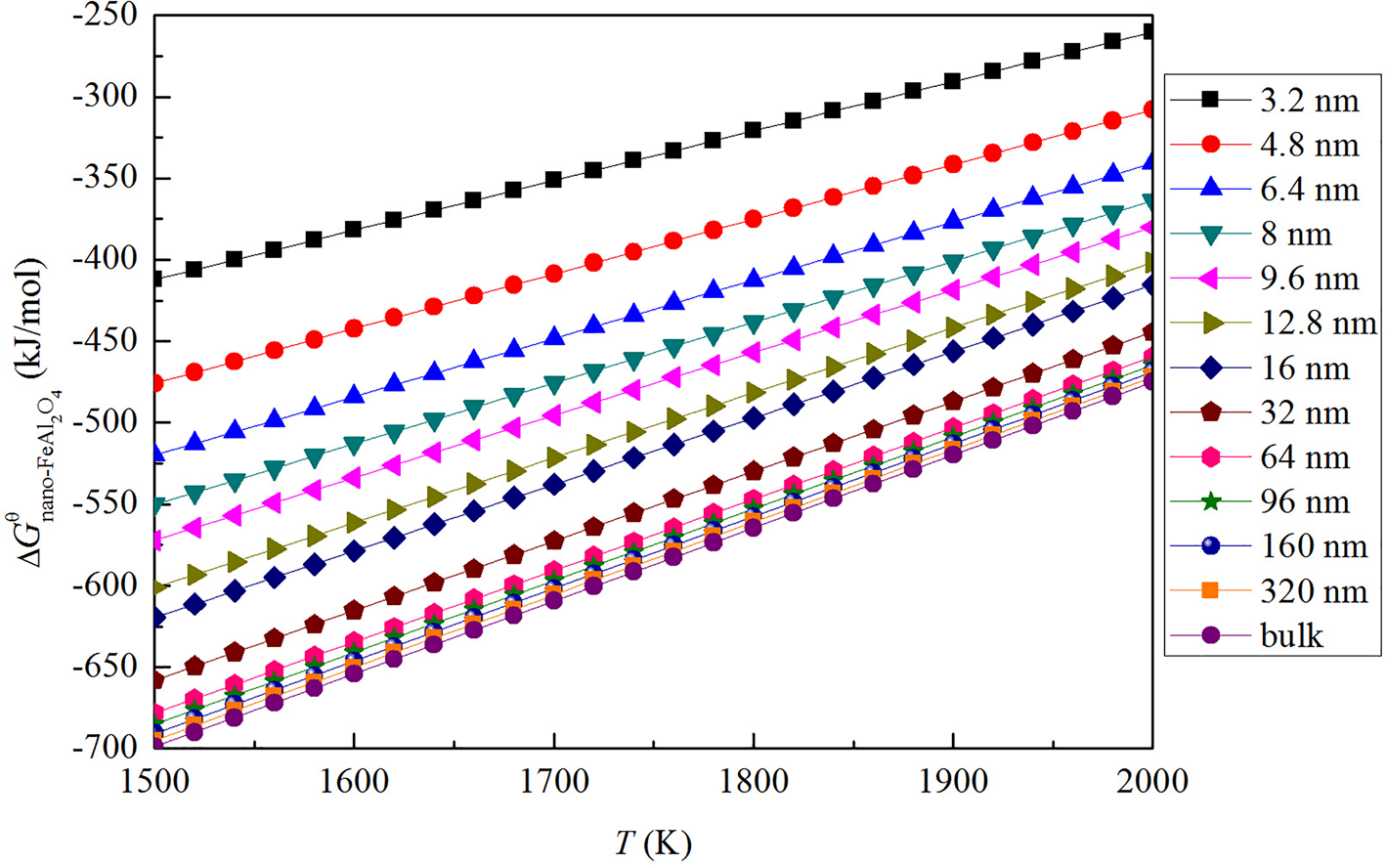
Gibbs free energy changes for the formation nano-FeAl_2_O_4_ in liquid iron.

Thus, the Gibbs free energy change for the formation of nano-FeAl_2_O_4_ in liquid iron $\Delta {\text{G}}_{{nano{ - }FeAl_{2} O_{4} }}^{\theta }$ can be calculated by Eqs. (5), (11) and (12). The Gibbs free energy changes for the formation of nano-FeAl_2_O_4_ in liquid iron are shown in Fig. 4. $\Delta {\text{G}}_{{nano{ - }FeAl_{2} O_{4} }}^{\theta }$ decreases with the increasing size and increases with the increasing temperature. This result implies that the thermodynamic driving energy for the formation of nano-FeAl_2_O_4_ increases gradually with the increasing of size and decreasing of temperature.

## Discussion

### Effect of products size on Al-deoxidation equilibrium thermodynamics

**Figure 5:**
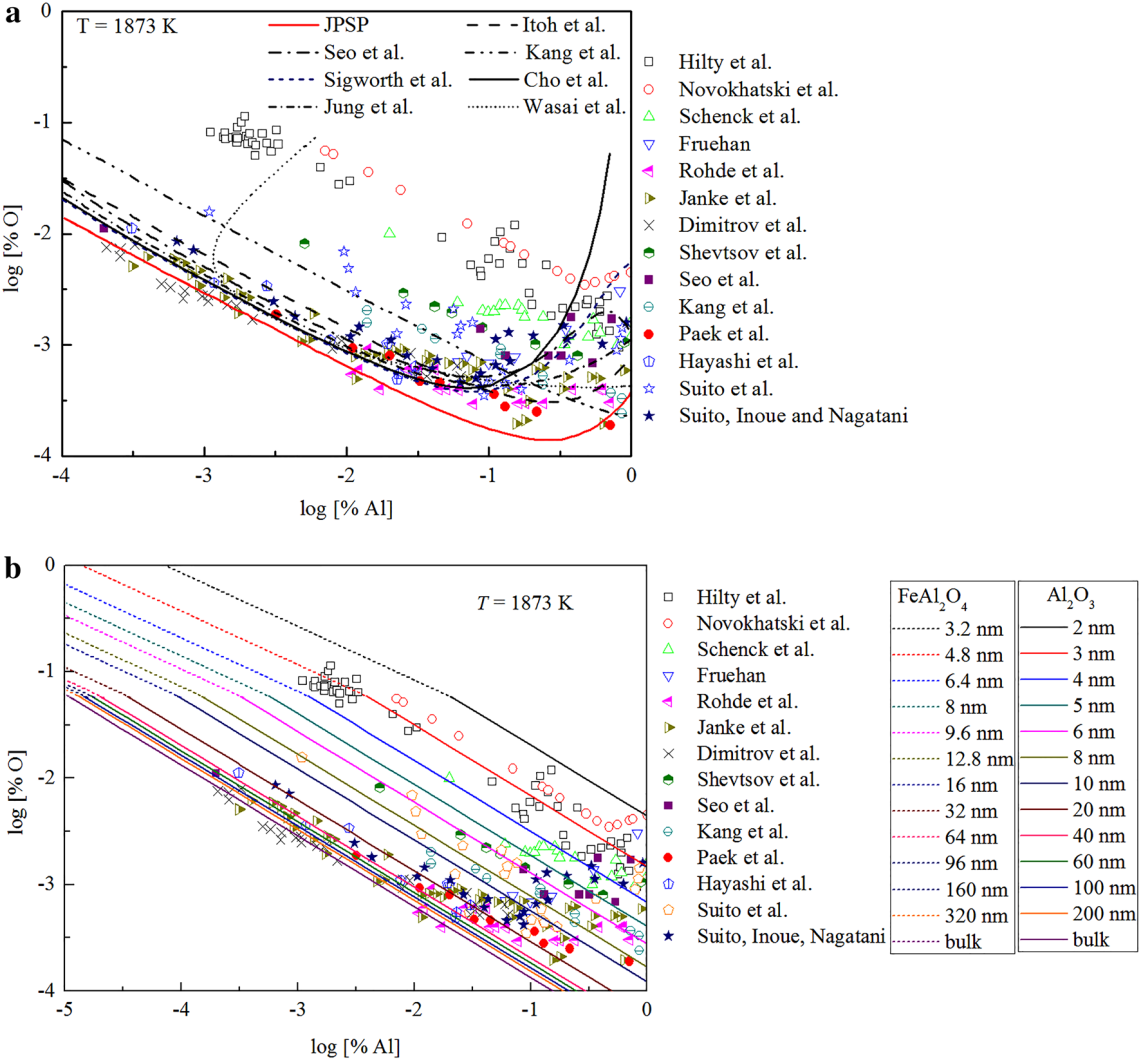
Thermodynamic curves of Al-deoxidation in liquid iron at 1873 K. (**a**) Equilibrium data obtained by previous work, (**b**) thermodynamic curves obtained by this work.

The equilibria for Al-deoxidation in liquid iron have been extensively investigated^12–25^. Table 2 lists the experimental conditions and methods of Al-deoxidation equilibrium in liquid iron at 1873 K. Most of the Al-deoxidation equilibria were measured by equilibrating liquid iron with [Al] and pure solid Al_2_O_3_ at 1873 K^12–14,18,19,22–25^. The Al-deoxidation experiments were generally carried out in alumina crucible by using rotating furnace^12^, resistance furnace^19,22^ and induction furnace^23–25^. Equilibrium concentration of [O] and [Al] were determined by analyzing the composition of experimental sample. The concentration of oxygen was generally analyzed by vacuum fusion method^12,13,18^, inert gas fusion method^14,19,22–25^, and neutron activation method^15^, while that of aluminum was obtained by wet-chemical analysis. On the other hand, in order to make the Al-deoxidation reaction more close to the final equilibrium state in liquid iron, Rohde et al.^16^, Suito et al.^19^, Paek et al.^25^ carried out their experiments with covered CaO–Al_2_O_3_ flux on the liquid iron. Meanwhile, the equilibrium oxygen potential in liquid iron was sometimes directly measured by using Electro Motive Force (EMF) technique^15,17,20,21^. It can be seen from Fig. 5a that the concentration of [O] decreases with the increasing [Al] when the concentration of [% Al] < 1%. It should be noted that, however, the difference among the concentrations of [O] reported by different researchers at the same concentration of [Al] is close to two order of magnitude.

**Table 2:** Experimental conditions and methods for Al-deoxidation equilibrium in liquid iron at 1873 K.

Authors	Experimental methods	[% Al]	Year
Hilty et al.^12^	Fe(l)–Al_2_O_3_(s)/V, rotating furnace	0.001–0.82	1950
Novokhatski et al.^13^	Fe(l)–Al_2_O_3_(s)/V	0.007–3.9	1966
Schenck et al.^14^	Fe(l)–Al_2_O_3_(s)/I	0.02–8.2	1970
Fruehan^15^	EMF/N	0.02–1.4	1970
Rohde et al.^16^	Fe(l)–CaO–Al_2_O_3_ slag	0.01–2.5	1971
Janke et al.^17^	EMF	0.0003–1.3	1976
Shevtsov et al.^18^	Fe(l)–Al_2_O_3_(s)/V	0.01–100	1981
Suito et al.^19^	Fe(l)–CaO–Al_2_O_3_ slag, Fe(l)–Al_2_O_3_(s)/I, resistance furnace	0.001–32.7	1991
Suito, Inoue, Nagatani^20^	EMF	0.0006–1.0	1992
Dimitrov et al.^21^	EMF	0.0001–1.2	1995
Seo et al.^22^	Fe(l)–Al_2_O_3_(s)/I, resistance furnace	0.0002–1.0	1998
Hanyashi et al.^23^	Fe(l)–Al_2_O_3_(s)/I, induction furnace	0.00031–0.0303	2008
Kang et al.^24^	Fe(l)–Al_2_O_3_(s)/I, induction furnace	0.01–10	2009
Paek et al.^25^	Fe(l)–CaO–Al_2_O_3_ slag, Fe(l)–Al_2_O_3_(s)/I, induction furnace	0.0027–100	2015

*V* vacuum fusion method, *N* neutron activation method, *I* inert gas fusion-infrared absorptiometry method.

In order to investigated the thermodynamic equilibrium rule of the Al-deoxidation reaction, most of the researchers^10,22,24,26–28^ established thermodynamic equilibrium curves based on the Wagner’s interaction parameter formalism. The thermodynamic equilibria curves obtained by various researchers are plotted as lines in Fig. 5a. At the concentration of [Al] < 0.1%, most of the equilibrium curves agree with their own experiments data and have the same trend that the concentration of [O] decreased with the increasing [Al]. When the [Al] content is greater than 0.1%, these equilibria curves show different shapes and be away from their experimental data to some extent. Later, the Modified Quasi chemical Model in the pair approximation in consideration of the strong short-range ordering mainly between Al, Fe and O have been used to establish the thermodynamic equilibrium relationship by Paek et al.^25^. It can be concluded from the Fig. 5a that there is not a same curve to describe all these experimental data of Al-deoxidation equilibria in liquid iron. Previous studies reported^29,30^ that the atoms of [Al] and [O] could not be independent randomly distributed, but have a strong tendency to form a kind of metastable phase, such as associated compound AlO, Al_2_O etc. In addition, Wang et al.^32–34^ suggested that the thermodynamic of Al-deoxidation in liquid iron is closely related with the size of alumina. Xiao et al.^35–37^ reported that the deoxidation thermodynamics of metal in liquid iron have depended on the structures and properties of reaction products. Hence, it can be concluded that there is a close relationship between the thermodynamics of Al-deoxidaiton reaction in liquid iron and metastable phase, such nano-Al_2_O_3_.

Wang et al.^32–34^ reported that the deoxidizers aluminum react with dissolved oxygen in molten steel to form various metastable alumina inclusions at first, and then the metastable alumina inclusion transform into stable crystal. However, the Al-deoxidation reaction in Fe–O–Al melt is very difficult to reach the thermodynamic equilibrium between bulk alumina and Fe–O–Al melt because of low supersaturation. Wasai et al.^31^ suggested that the small alumina nuclei are suspended in liquid iron, and this is one reason for the presence of excess oxygen in liquid iron. Later, Wasai et al.^8^ found a series of nano-alumina by hold their Al-deoxidation experiments at 1873 K in alumina crucible. In their experiments, the Al-deoxidized iron was maintained at 1873 K for a certain time (1, 5, 15, 30, 60 min), and the Al-deoxidized iron was solidified at 3 different cooling speed. The minimum diameter of alumina inclusions observed in their work is in the range from a few nm to 10 nm. This result indicates that the Al-deoxidation products are nanoscale alumina even 60 min after Al-deoxidation in the liquid iron.

As the reaction proceeds, the thermodynamic driving force of Al-deoxidation in liquid iron decreased gradually with the decreasing supersaturation ratio^31^. Therefore, it is very difficult for the nano-Al_2_O_3_ (or nano-FeAl_2_O_4_) to grow up into the final bulk crystal at the later period of Al-deoxidation. As a result, the nano-Al_2_O_3_ (or nano-FeAl_2_O_4_) are not large enough to float upward, and can appear as the structural units in Fe–O–Al melt and may remain as suspending inclusions in the melt for a long time. Therefore, the reaction products of Al-deoxidation in liquid iron may be various nano-Al_2_O_3_ and nano-FeAl_2_O_4_ inclusions in many cases.

Based on the Gibbs free energy changes for the formation of different size of Al_2_O_3_ and FeAl_2_O_4_ in liquid iron at 1873 K, the thermodynamic curves for the Al-deoxidation in liquid iron can be obtained. As shown in Fig. 5b, the thermodynamic curves for different size of alumina (solid line) and hercynite (dash line) means that the product of Al-deoxidation in liquid iron can be different size of alumina and hercynite. It should be noted that the diameter of FeAl_2_O_4_ is 1.6 times that of α-Al_2_O_3_ when they have the same number of aluminum atoms. This is because of the “molecule densities” of FeAl_2_O_4_ is 1.6 times as that of Al_2_O_3_ according to the calculation result. It can be seen from Fig. 5b, most of the Al-deoxidation experimental data are covered by the region between the bulk-alumina (or bulk-hercynite) equilibrium curve and the thermodynamic curves of 2 nm alumina (or 3.2 nm hercynite). Such facts indicate that the Al-deoxidation product should not be bulk-crystal but in nanoscale in most of those equilibria experiments. It can be concluded that the Al-deoxidation experiments by various researchers are in different thermodynamic state. These different thermodynamic states in their experiments may lead by the different experimental conditions. In addition, the nano-alumina (or nano-hercynite) thermodynamic curves are close to the bulk-alumina (or bulk-hercynite) equilibrium curve gradually with the increase of size of Al-deoxidation products. It indicates that the Al-deoxidation reaction is gradually close to the equilibrium between bulk alumina (or bulk-hercynite) and liquid iron with the increasing of Al-deoxidation products size during growth process. In other words, the equilibria experiments of various researchers could be in a state away from the final equilibrium between bulk alumina and liquid iron to some extent.

### Thermodynamic coexisting point of the formation of Al_2_O_3_ and FeAl_2_O_4_ in liquid iron

**Figure 6:**
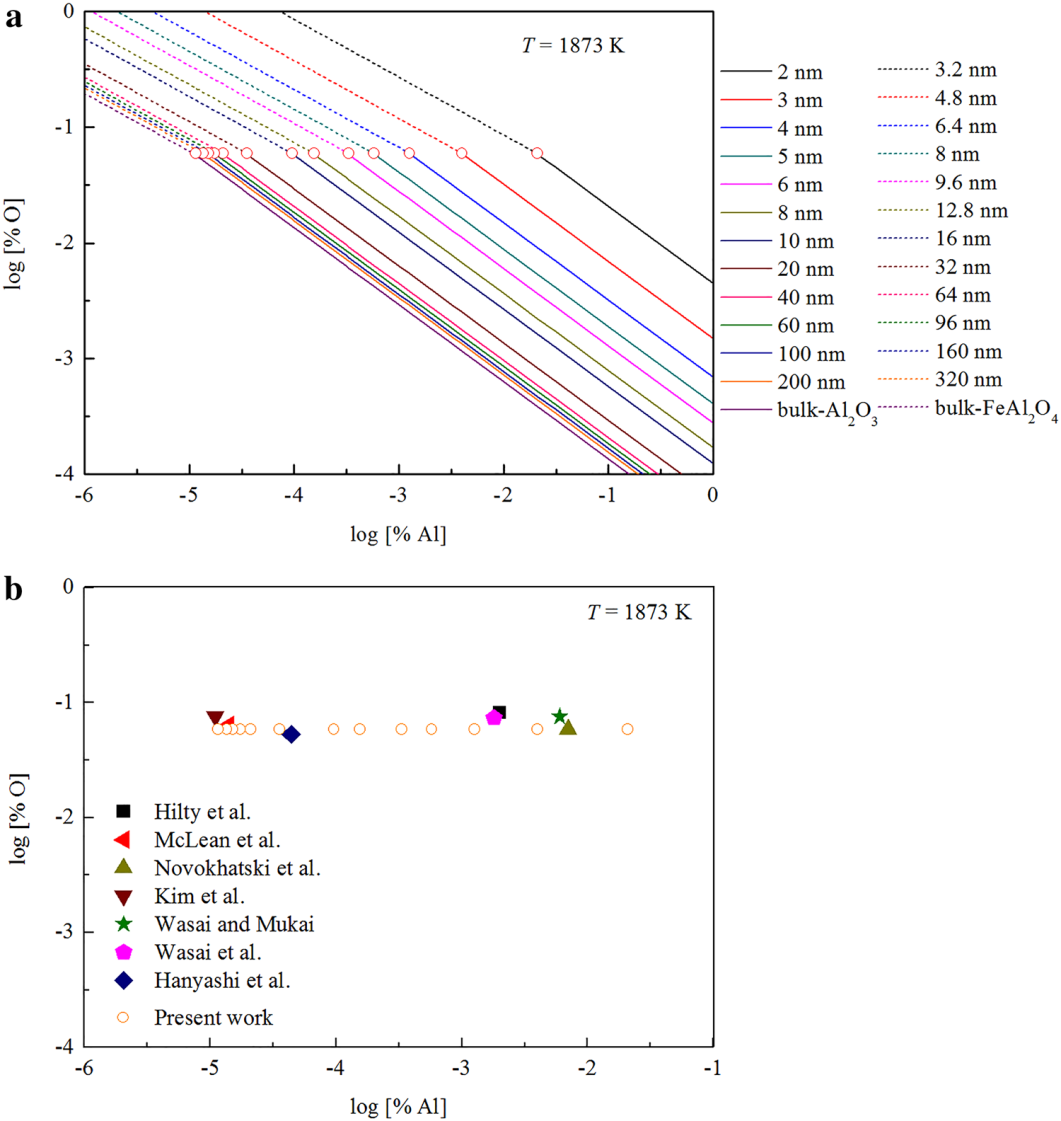
Thermodynamic coexisting points for the formation of Al_2_O_3_ and FeAl_2_O_4_ in liquid iron at 1873 K. (**a**) The thermodynamic coexisting points obtained by this work, (**b**) comparison between the present thermodynamic coexisting points and previous equilibrium coexisting point.

As shown in Fig. 6a, the intersection points of thermodynamic curves for alumina and corresponding hercynite (hercynite have the same number of aluminum atoms with alumina) are the thermodynamic coexisting points, while the intersection of thermodynamic curves for bulk alumina and bulk hercynite is the equilibrium coexisting point. The products in these points are both alumina and hercynite, and their size increase from right to left.

As known, there are three phases (liquid iron, hercynite and alumina) in Fe–Al–O ternary system, so the freedom degree is zero if this ternary system is under the specified pressure and temperature. Consequently, there is only one equilibrium coexisting point when the liquid iron equilibrated with both alumina and hercynite. Such an equilibrium coexisting point at 1873 K has been measured by several researchers^11–13,23,31,47,48^. McLean et al.^11^ measured the oxygen content of liquid iron in equilibrium with Al_2_O_3_ and FeO·Al_2_O_3_ at temperatures between 1823 and 2023 K, and reported the coexisting point to be 6.23 × 10^–2^ [% O], 1.4 × 10^–5^ [% Al] at 1873 K. Kim et al.^47^ measured the equilibrium at the temperature between 1813 and 1983 K, and reported the coexisting point to be 7.4 × 10^–2^ [% O], 1.1 × 10^–5^ [% Al] at 1873 K. Hilty et al.^12^ reported the coexisting point (8 × 10^–2^ [% O], 2 × 10^–3^ [% Al]) by melting oxidized electrolytic Fe in alumina crucible or adding FeO to Fe-Al melt at 1873 K. Meanwhile, Wasai and Mukai^31^ reported their coexisting point to be 7.4 × 10^–2^[% O], 6.3 × 10^–3^ [% Al] using an associated solution model at 1873 K. It should be noticed that the oxygen concentrations of coexisting point are close to each other, but the difference about the aluminum concentrations is more than two orders magnitude in these experiments, as shows in the Fig. 6b.

These equilibria experiments may be in the different thermodynamic states due to different experimental conditions. Thus, the deoxidation products maybe a series of different size nanoscale hercynite and alumina in their three-phase equilibrium experiments. These different values of equilibrium coexisting points by various researchers may have a close relationship with the size of Al-deoxidation products. In some case of experiments, the three equilibrium coexisting phases in Fe–O–Al melt could be nano-alumina, nano-hercynite and liquid iron. Therefore, the coexisting points may be different to each other. As can be seen from Fig. 6b, all the predicted coexisting points are located at the same horizontal line. The concentrate of [Al] at the coexisting point for 2 nm Al_2_O_3_ is more than three orders of magnitude less than that for bulk Al_2_O_3_. These results agree well with the previous experimental data^11–13,23,31,47,48^. Such a fact indicates that it needs more time for the nano-Al_2_O_3_ (or nano-FeAl_2_O_4_) to grow up into bulk-Al_2_O_3_ (or bulk-FeAl_2_O_4_) in some cases. It suggested that these coexisting points measured by various experiments could be in different thermodynamic state. Most of the products of three-phase equilibrium in Fe–O–Al melt are not bulk-Al_2_O_3_ and bulk-FeAl_2_O_4_ but also various different size nano-Al_2_O_3_ and nano-FeAl_2_O_4_. Thus, these coexisting points proved again that many previous experiments are close to the final equilibrium, but not reach the final equilibrium.

## Conclusions

The conclusions are as follows:

1. The surface of nano-alumina or nano-hercynite plays an important part in their thermodynamic properties. The Gibbs free energy changes for the formation of nano-Al_2_O_3_ and nano-FeAl_2_O_4_ decrease with the increasing size and increase with the increasing temperature. The Gibbs free energy changes for transformation of nano-Al_2_O_3_ into bulk-Al_2_O_3_ increase with the increasing size and temperature.
2. The published experimental data for Al-deoxidation equilibria in liquid iron are scattered across the region between the thermodynamic curve of nano-alumina (or nano-hercynite) and the equilibrium curve of bulk-alumina (or bulk-hercynite). The thermodynamic coexisting points about Al_2_O_3_ and FeAl_2_O_4_ in liquid iron are in a straight line and coincide with the various previous data.
3. Many of the previous Al-deoxidation experiments are close to the final thermodynamic equilibrium between bulk alumina or hercynite and liquid iron but not reach the final thermodynamic equilibrium because their partial product is nano-alumina or nano-hercynite.
